# Unravelling the Itch: Cross‐Sectional Analysis of Pruritus Patterns in the PicnicHealth Primary Biliary Cholangitis Registry

**DOI:** 10.1111/liv.70809

**Published:** 2026-09-09

**Authors:** Cynthia Levy, Daniel C. Gibbons, Sophia Abner, Haley S. Friedler, Dhirishiya P, Harini Vadlamudi, Veronika Earley‐Valovic, Anna Coutinho, Kaitlin Hagan, Kejsi Begaj, Anna Halliday

**Affiliations:** ^1^ Digestive Health and Liver Diseases, Miller School of Medicine University of Miami Miami Florida USA; ^2^ Schiff Center for Liver Diseases University of Miami Miami Florida USA; ^3^ GSK London UK; ^4^ PicnicHealth San Francisco California USA; ^5^ GSK Bangalore India; ^6^ GSK Collegeville Pennsylvania USA; ^7^ GSK Boston Massachusetts USA

**Keywords:** alkaline phosphatase, primary biliary cholangitis, pruritus, quality of life

## Abstract

**Background & Aims:**

Pruritus in primary biliary cholangitis (PBC) worsens patients' health‐related quality of life (HRQoL). Serum alkaline phosphatase (ALP), a biomarker of PBC activity, has an unclear relationship with pruritus severity.

**Methods:**

This retrospective, cross‐sectional analysis utilized data from participants with confirmed PBC diagnoses, from the PicnicHealth PBC registry, with ≥ 1 completed PBC‐40 questionnaire (most recent was used). The primary analysis included participants with a qualifying ALP measurement. Participants were categorized by pruritus severity per PBC‐40 itch domain (clinically significant [CS] pruritus [score ≥ 7], or non‐CS pruritus [score = 1–6]); serum ALP (normal [ALP ≤ 1 × upper limit of normal; ULN], mildly elevated [ALP > 1–< 1.67 × ULN], or high [ALP ≥ 1.67 × ULN]; primary analysis). Primary outcome: proportion of participants in each pruritus severity subgroup by ALP category. Secondary outcome (all participants with PBC‐40): HRQoL by pruritus severity.

**Results:**

Overall, 232 participants completed ≥ 1 PBC‐40 questionnaire; 141 had a qualifying ALP measurement. Most participants with pruritus were female (97%) and white (93%); 53% had CS pruritus. 23% (13/57), 32% (17/53) and 52% (16/31) of participants with normal, mildly elevated and high ALP, respectively, reported CS pruritus. Participants with CS pruritus had significantly higher scores across non‐itch PBC‐40 domains (*p* < 0.05) versus non‐CS pruritus and greater odds of having CS PBC‐40 domain scores versus no CS pruritus (*p* < 0.0001; odds ratio [95% CI]: Symptoms 7.08 [3.89, 12.90]; Fatigue 9.51 [4.71, 19.22]; Emotional 2.67 [1.50, 4.76]; Social 6.14 [3.47, 10.88]; Cognitive 4.03 [2.33, 6.96]).

**Conclusions:**

CS pruritus is common, present in patients irrespective of ALP levels and significantly impacts HRQoL.

## Key Points

- Pruritus has a negative impact on health‐related quality of life (HRQoL) in patients with primary biliary cholangitis (PBC). There is an unclear relationship between serum alkaline phosphatase (ALP; a biomarker of PBC activity) and pruritus severity.
- This retrospective, cross‐sectional analysis of patients with PBC found that clinically significant pruritus was common and present in patients irrespective of ALP levels.
- Patients with clinically significant pruritus had worse HRQoL than those without clinically significant pruritus.
- Every patient with PBC should be assessed for pruritus and its impact on HRQoL, regardless of biochemical disease control.

## Lay Summary

- Itch in primary biliary cholangitis (PBC) worsens patients' quality of life. Alkaline phosphatase (ALP) is an enzyme found in the blood, which is often higher in patients with PBC; however, its relationship with the severity of itch is unclear.
- This study showed that itch is common, can be present in patients with PBC irrespective of ALP levels and can strongly impact quality of life.
- Doctors should assess every patient with PBC for itch and its effect on quality of life, regardless of liver test results and involve patients in the decision making for better disease management, including treatment needs specifically for itch.

**Abbreviations**
ALP: alkaline phosphatase
CS: clinically significant
HRQoL: health‐related quality of life
PBC: primary biliary cholangitis
SD: standard deviation
UDCA: ursodeoxycholic acid
ULN: upper limit of normal

## 1 Introduction

Cholestatic pruritus affects 55%–89% of patients with primary biliary cholangitis (PBC) [1, 2, 3, 4]. Patients with PBC and clinically significant pruritus have significantly worse health‐related quality of life (HRQoL) than patients with mild or no pruritus [2, 3], as assessed by the PBC‐40, a psychometrically validated HRQoL measurement [5]. Recently, the ITCH‐E study investigated the impact of pruritus on HRQoL, activity and work productivity and identified that, in addition to worse HRQoL, greater pruritus severity in patients with PBC is also associated with reduced productivity [6]. Furthermore, pruritus has been shown to negatively impact emotional wellbeing and mental health, contribute to social isolation and interfere with sleep, which in turn contributes to fatigue [1, 7, 8]. Sleep disturbance due to pruritus has also been shown to correlate with depression and anxiety and worse disease‐specific QoL [9].

Alkaline phosphatase (ALP) is typically elevated in patients with PBC, and guidelines recommend the measurement of ALP for diagnosis and assessment of treatment response [10]. However, patients with PBC can experience clinically significant pruritus despite normal ALP levels; therefore, the unclear relationship between PBC activity (as measured by serum ALP) and pruritus severity warrants further investigation [3, 11, 12, 13]. Serum ALP has previously been identified as an independent risk factor of pruritus in PBC [4], with a correlation between higher ALP and more severe pruritus also observed when compared to those with mild or no pruritus [1, 2]. However, another study reported no association between ALP and the PBC‐40 Itch domain [13]. Analysis of the baseline characteristics of patients with PBC included in the GLISTEN Phase 3 trial, which enrolled patients with PBC and moderate‐to‐severe pruritus, also identified that 52% of patients had ALP < 1.67 × the upper limit of normal (ULN), indicating that pruritus can be present despite disease control [11].

With increased availability of second‐line therapies for PBC and the development of newer therapies, more patients are achieving a biochemical response. However, laboratory measures indicating biochemical response may not necessarily equate to a reduction in pruritus severity, and the impact of any ongoing pruritus may still be affecting patient QoL. This study aimed to utilize patient‐reported outcomes to assess the prevalence of pruritus across subgroups of patients with PBC defined by ALP levels, and further explore the impact of PBC‐related pruritus and pruritus‐related sleep disturbance on HRQoL, using data from the PicnicHealth PBC registry.

## 2 Methods

### 2.1 Study Design

This was a retrospective, cross‐sectional analysis of participants living in the US who were enrolled in the PicnicHealth PBC registry [3]. Eligible participants were recruited from early January 2021 to August 2023 through patient advocacy groups, affiliate partners and digital marketing campaigns. Neither the participants nor the advocacy groups/representatives were aware that the purpose of the study was to evaluate pruritus, and pruritus presence and severity were not known prior to enrollment. Participants had a confirmed diagnosis of PBC and at least one completed PBC‐40 assessment. The index date was defined as the date of a participant's most recent PBC‐40 measure, with or without an available ALP measurement. Participants receiving obeticholic acid prior to index were excluded from the primary ALP analysis; use of other second‐line or investigational therapies (e.g., fibrates/PPAR agonists) was permitted.

On average, 7–8 years of retrospective participant data were collected, along with prospectively updated medical records, using PicnicHealth's natural language processing and human‐reviewed machine learning‐enabled platform to generate a de‐identified patient‐level database [14]. Medical records were received in any format from the facility or provider. Data were abstracted from both structured data, such as medication lists, coded past medical history lists, vital signs, laboratory data, as well as unstructured narrative text (i.e., clinical notes) from medical records.

Data curation followed a structured, ‘human‐in‐the‐loop’ abstraction process in which trained clinical research coordinators (with relevant medical or clinical backgrounds) reviewed medical records and extracted data. Annotators underwent study‐specific training, including instruction on abstraction completion guidelines and were required to pass an assessment prior to performing data abstraction. Data abstraction was supported by machine learning–based predictions; however, all extracted data were reviewed and validated by human annotators, who confirmed or corrected model outputs based on source documentation. Clinical concepts were aligned to standard medical ontologies (i.e., common terminologies, vocabularies and coding schemes to facilitate data analysis). To support data quality, standardized abstraction guidelines and structured data entry were used. In addition, programmatic quality checks were applied to identify inconsistencies, missing data and implausible values within the dataset. In order to collect all available patient medical records, already obtained records and claims data were assessed to identify additional missing visits or providers. The final data cut‐off for this study was October 2023.

Sociodemographic characteristics and ALP measures were extracted from medical records. Participants were invited to complete the PBC‐40 questionnaire at enrollment and then quarterly thereafter. The PBC‐40 questionnaire (with 4‐week recall) is a psychometrically validated questionnaire designed to measure HRQoL, which comprises 40 questions across six domains of health (General Symptoms, Itch, Fatigue, Cognition, Social and Emotional). Each question is scored on a scale of 0 (when questions are not applicable) or 1 (least impact) to 5 (greatest impact). Thresholds for clinical significance (and the minimum and maximum possible score) for each of the PBC‐40 domains are: Symptoms: 18 (6–35), Fatigue: 33 (11–55), Emotional: 12 (3–15), Social: 32 (8–50), Cognitive: 18 (6–30) and Itch: 7 (0–15) [2, 15].

To be eligible for inclusion in the primary ALP analysis, participants required a qualifying ALP measure (an ALP measure recorded within a 6‐month window [i.e., +/−3 months] around the participant's most recent PBC‐40 assessment for the primary analysis and a 12‐month window [i.e., +/−6 months] for the sensitivity analysis). The most recent PBC‐40 questionnaire was selected for each patient to enable comparison of itch severity across ALP categories and optimize temporal alignment between patient‐reported outcomes and laboratory measurements.

### 2.2 Participant Categorization

Participants were categorized according to pruritus severity as assessed by the PBC‐40 Itch domain: no pruritus (score = 0), any pruritus (score ≥ 1), non‐clinically significant pruritus (score = 1–6), or clinically significant pruritus (score ≥ 7) [15].

Eligible participants were also categorized according to serum ALP levels: normal (ALP ≤ 1 × ULN); mildly elevated (ALP > 1 to < 1.67 × ULN); or high (ALP ≥ 1.67 × ULN).

### 2.3 Outcomes

The primary outcome was to describe the proportion of participants in each pruritus severity subgroup by ALP category. The key secondary outcome was to describe HRQoL across non‐itch PBC‐40 domains (Symptoms, Fatigue, Cognitive, Emotional and Social), with participants categorized by pruritus severity. The exploratory outcome was to describe HRQoL across non‐itch PBC‐40 domains, with participants classified by response to the PBC‐40 question: ‘in the past 4 weeks, itching never/rarely/sometimes/most of the time/always disturbed my sleep’.

### 2.4 Data and Statistical Analysis

Unless otherwise specified, analyses were descriptive in nature and were reported as mean, standard deviation (SD), medians, interquartile range, minimums, maximums. Chi‐squared tests were used to assess independence between PBC‐40 itch severity and ALP categories. Individual PBC‐40 item scores were summed to give a total domain score. Zero scores (indicating that the question was not applicable) were included within the summary statistics. Demographic and clinical characteristics, including ursodeoxycholic acid (UDCA) use, were assessed across both ALP and PBC‐40 Itch domain categories.

Odds ratios for the presence of clinically significant non‐itch PBC‐40 domain scores (Symptoms, Fatigue, Cognitive, Emotional and Social) [15] were determined for participants with and without clinically significant pruritus (≥ 7 vs. < 7 on the itch domain scores). Statistical comparisons between pruritus severity categories were made where statistical power was ≥ 80% for a two‐sided significance of 0.05. Analyses were not adjusted for baseline participant characteristics.

## 3 Results

### 3.1 Participant Population

A total of 141 participants had both a PBC‐40 assessment and a qualifying ALP measurement and were included in the analysis of the primary outcome. A greater number of participants (*N* = 232) had ≥ 1 PBC‐40 questionnaire completed and were included in the analysis of the key secondary outcome. Of those, 208 participants were included in the analysis of the exploratory outcome; the 24 participants not included in this analysis selected ‘no itch/does not apply’ in response to the PBC‐40 question: ‘in the past 4 weeks, itching never/rarely/sometimes/most of the time/always disturbed my sleep’.

Demographics and clinical characteristics of participants with PBC according to ALP category are summarized in Table 1. There were slightly fewer participants with high ALP (31/141, 22%) than participants with mildly elevated or normal ALP (53/141 [38%] and 57/141 [40%], respectively). All participants included in the ALP analysis had a history of UDCA use, regardless of ALP category (Table 1). Demographics were numerically fairly balanced across all groups, irrespective of ALP status.

**TABLE 1:** Demographic and clinical characteristics of participants with primary biliary cholangitis, by ALP category (*N* = 141).

	All participants (*N* = 141)	Normal ALP^a^ (*n* = 57)	Mildly elevated ALP^b^ (*n* = 53)	High ALP^c^ (*n* = 31)
Female sex, *n* (%)	138 (97.9)	56 (98.3)	53 (100.0)	29 (93.6)
Years since PBC diagnosis (to enrollment date), mean (SD)	5.7 ± 6.0	6.8 ± 6.0	4.6 ± 4.3	5.6 ± 8.1
Age at PBC diagnosis (years), mean (SD)	49.6 ± 9.6	49.7 ± 9.5	51.0 ± 9.1	47.2 ± 10.6
Age at enrollment (years), mean (SD)	55.8 ± 10.6	57.0 ± 10.7	56.1 ± 9.2	53.2 ± 12.4
Age at PBC‐40 completion date^d^ (years), mean (SD)	56.5 ± 10.6	57.7 ± 10.5	56.8 ± 9.3	53.8 ± 12.5
Race, *n* (%)				
Black or African American	3 (2.1)	2 (3.5)	0	1 (3.2)
White	131 (92.9)	53 (93.0)	48 (90.6)	30 (96.8)
More than one race	4 (2.8)	1 (1.8)	3 (5.7)	0
Unknown	3 (2.1)	1 (1.8)	2 (3.8)	0
Ethnicity, *n* (%)				
Hispanic or Latino	10 (7.1)	2 (3.5)	5 (9.4)	3 (9.7)
Neither Hispanic or Latino	128 (90.8)	53 (93.0)	48 (90.6)	27 (87.1)
Unknown	3 (2.1)	2 (3.5)	0	1 (3.2)
Charlson Comorbidity Index, mean (SD)	2.4 ± 1.4	2.6 ± 1.5	2.2 ± 1.4	2.3 ± 0.9
PBC Stage, *n* (%)				
Stage 1	31 (22.0)	12 (21.1)	15 (28.3)	4 (12.9)
Stage 2	15 (10.6)	4 (7.0)	4 (7.5)	7 (22.6)
Stage 3	6 (4.3)	2 (3.5)	2 (3.8)	2 (6.5)
Stage 4	3 (2.1)	0	2 (3.8)	1 (3.2)
Missing	89 (63.1)	39 (68.4)	33 (62.3)	17 (54.8)
Ever diagnosed with cirrhosis, *n* (%)	64 (45.4)	28 (49.1)	24 (45.3)	12 (38.7)
UDCA use (ever), *n* (%)	141 (100)	57 (100)	53 (100)	31 (100)

Abbreviations: PBC; primary biliary cholangitis; UDCA, ursodeoxycholic acid; ULN, upper limit of normal.

^a^ ALP, ≤ 1 × ULN.

^b^ ALP > 1 to < 1.67 × ULN.

^c^ ALP ≥ 1.67 × ULN.

^d^ Most recent PBC‐40 completion within the data collection window.

Demographics and clinical characteristics of participants with PBC categorized by pruritus severity are summarized in Table 2. The majority of participants had pruritus (score ≥ 1; 210/232 [91%]); of those, more than half had clinically significant pruritus (score ≥ 7; 122/210 [58%]). Participants with any pruritus had higher mean ALP than participants with no pruritus, and the same trend was observed in clinically significant pruritus versus non‐clinically significant pruritus. Nearly all participants included in the analyses had a history of ever using UDCA (231/232 [99.6%]). Due to the large proportion of missing data, descriptive analyses of PBC stage and pruritus have not been included.

**TABLE 2:** Demographic and clinical characteristics of participants with primary biliary cholangitis, by pruritus severity (*N* = 232).

	No pruritus (*n* = 22)	Any pruritus (*n* = 210)	Non‐clinically significant pruritus (*n* = 88)	Clinically significant pruritus (*n* = 122)
Female sex, *n* (%)	22 (100.0)	204 (97.1)	85 (96.6)	119 (97.5)
Years since PBC diagnosis (to enrollment date), mean (SD)	6.2 ± 5.5	6.0 ± 5.8	5.9 ± 6.3	6.0 ± 5.5
Age at PBC diagnosis, mean (SD)	52.4 ± 9.4	48.4 ± 10.0	50.3 ± 10.6	46.9 ± 9.3
Age at enrollment, mean (SD)	59.0 ± 10.2	54.8 ± 10.7	56.7 ± 11.3	53.4 ± 10.1
Age at PBC‐40 completion date^a^ (years), mean (SD)	60.4 ± 10.2	56.2 ± 10.8	58.2 ± 11.3	54.8 ± 10.1
ALP^b^, U/L mean (SD)	125.3 ± 50.5	166.2 ± 123.3	136.2 ± 63.1	188.3 ± 149.5
Race, *n* (%)				
American Indian or Alaska Native	0	2 (1.0)	0	2 (1.6)
Black or African American	0	3 (1.4)	1 (1.1)	2 (1.6)
White	19 (86.4)	195 (92.9)	86 (97.7)	109 (89.3)
More than one race	1 (4.6)	7 (3.3)	0	7 (5.7)
Unknown	2 (9.1)	3 (1.4)	1 (1.1)	2 (1.6)
Ethnicity, *n* (%)				
Hispanic or Latino	2 (9.1)	14 (6.7)	5 (5.7)	9 (7.4)
Neither Hispanic or Latino	18 (81.8)	193 (91.9)	81 (92.0)	112 (91.8)
Prefer not to say	2 (9.1)	3 (1.4)	2 (2.3)	1 (0.8)
Charlson Comorbidity Index, mean (SD)	2.3 ± 1.4	2.2 ± 1.3	2.2 ± 1.1	2.2 ± 1.5
PBC Stage, *n* (%)				
Stage 1	2 (9.1)	34 (16.2)	17 (19.3)	17 (13.9)
Stage 2	2 (9.1)	29 (13.8)	11 (12.5)	18 (14.8)
Stage 3	1 (4.5)	10 (4.8)	4 (4.5)	6 (4.9)
Stage 4	2 (9.1)	4 (1.9)	0	4 (3.3)
Missing	15 (68.2)	133 (63.3)	56 (63.6)	77 (63.1)
Ever diagnosed with cirrhosis, *n* (%)	13 (59.1)	100 (47.6)	38 (43.2)	62 (50.8)
UDCA use (ever), *n* (%)	22 (100)	209 (99.5)	87 (98.9)	122 (100)

*Note:* No pruritus, score = 0; any pruritus, score ≥ 1; non‐clinically significant pruritus, score 1–6; clinically significant pruritus, score ≥ 7.

Abbreviations: PBC; primary biliary cholangitis; UDCA, ursodeoxycholic acid.

^a^ Most recent PBC‐40 completion within the data collection window.

^b^ ALP result captured closest to PBC‐40 completion.

### 3.2 Primary Outcome

**FIGURE 1:**
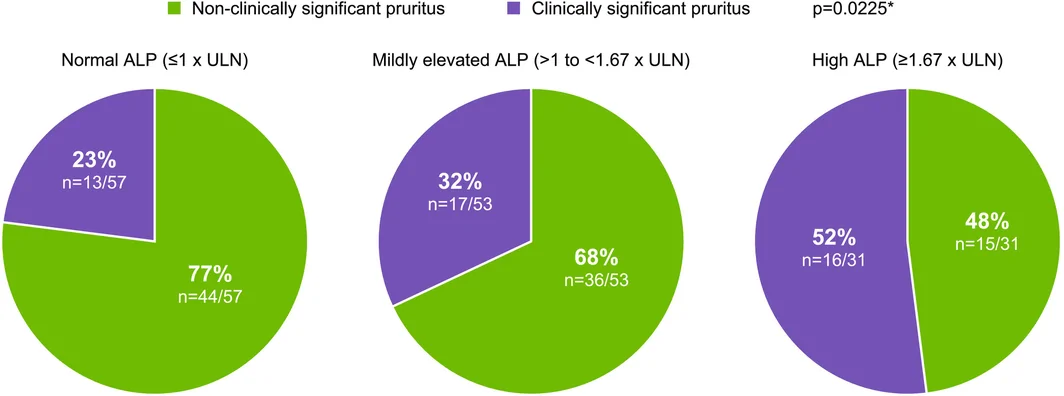
Proportion of participants with clinically significant pruritus by ALP category. *p values from Chi‐squared test to assess independence between PBC‐40 Itch severity and ALP levels. ULN, upper limit of normal.

The proportion of participants with clinically significant pruritus increased with higher ALP (*p* = 0.0225; Figure 1). However, clinically significant pruritus was observed across all ALP categories: 23% (13/57) of participants with normal ALP, 32% (17/53) of participants with mildly elevated ALP and 52% of participants with high ALP (16/31; Figure 1). Similar trends were observed in the sensitivity analysis, although the association between ALP and itch severity categories was not statistically significant (*p* = 0.0947).

### 3.3 Secondary and Exploratory Outcomes

**FIGURE 2:**
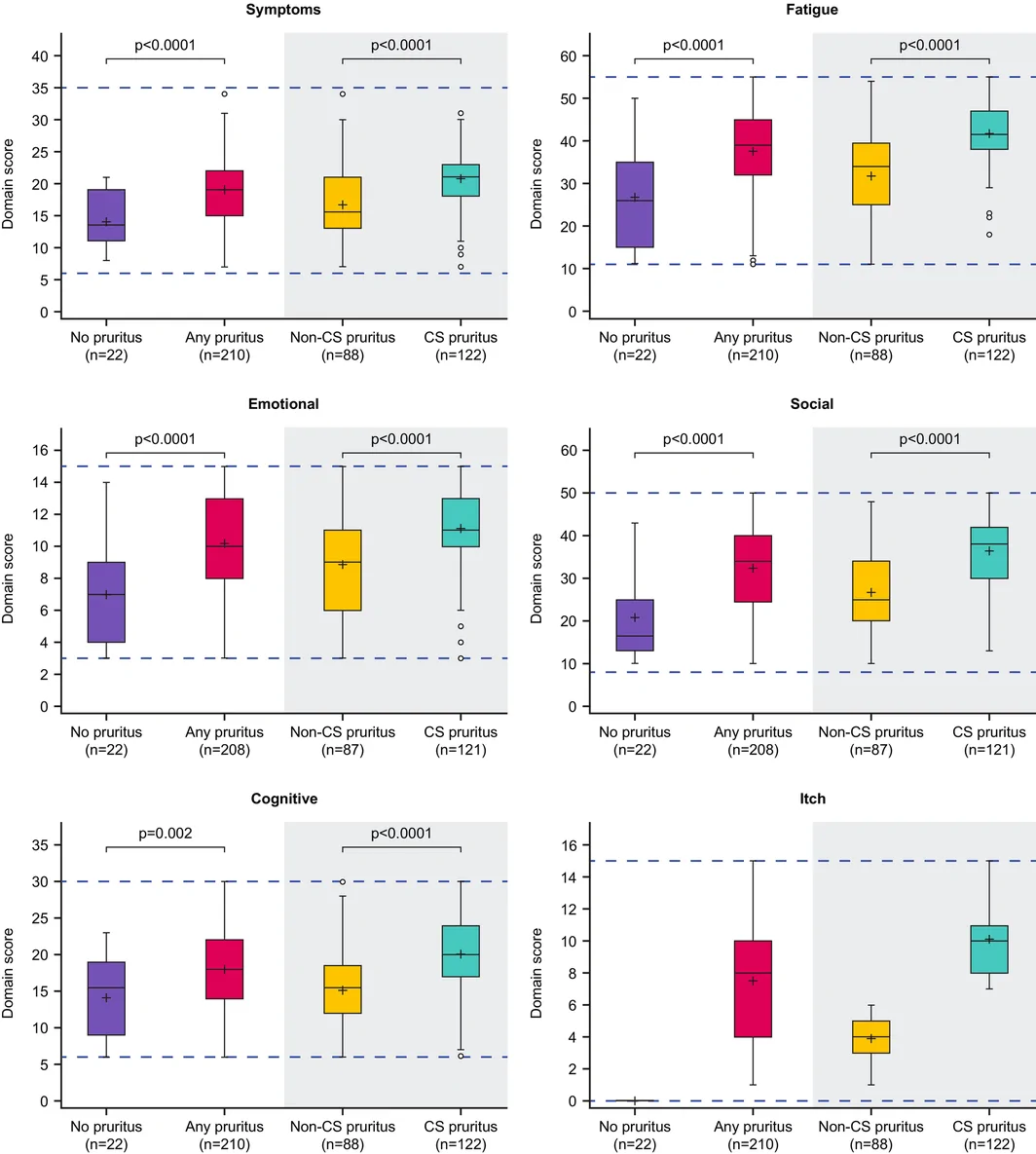
Average score for primary biliary cholangitis‐40 domains by presence and severity of pruritus. Thresholds for clinical significance (and the minimum and maximum possible score) for each of the domains were: Symptoms: 18 (6–35), Fatigue: 33 (11–55), Emotional: 12 (3–15), Social: 32 (8–50), Cognitive: 18 (6–30) and Itch: 7 (0–15). Scores of zero indicate a statement was not applicable or that no itch was associated with that question, and therefore zero scores were included in the analysis. Dotted blue lines represent the minimum and maximum possible scores for each domain. Median depicted by the black line, upper and lower quartiles by upper/lower box, min/max as error bars. Values that were more than 1.5 times the interquartile range away from the box were considered outliers and are shown as circles. Cross represents the mean. Pruritus categories are not mutually exclusive (i.e., the any pruritus category comprises non‐CS pruritus and CS pruritus categories). Statistical analyses compared the ‘no pruritus’ to the ‘any pruritus’ cohorts and the ‘non‐CS itch’ with the ‘CS itch’ cohorts; these comparisons were only completed for the non‐itch domains of PBC‐40. The ‘any pruritus’ cohort comprised the ‘non‐CS itch’ and ‘CS itch’ cohorts. CS, clinically significant; PBC, primary biliary cholangitis.

**FIGURE 3:**
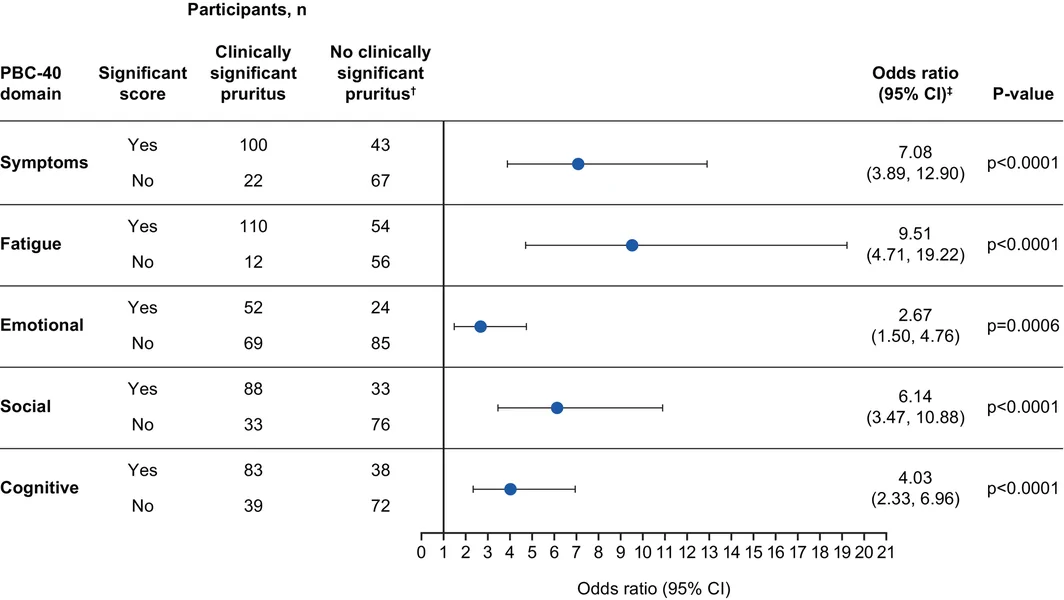
Odds ratios for having clinically significant primary biliary cholangitis‐40 score in those with versus without clinically significant pruritus. ^†^This includes participants with non‐clinically significant itch and no itch. ^‡^Odds ratios for the presence of clinically significant PBC‐40 domain scores, based on the cut‐offs recommended by the developers of the PBC‐40 [15], determined for participants with and without clinically significant pruritus (i.e., ≥ 7 vs. < 7 on the itch domain score). Thresholds for clinical significance (and the minimum and maximum possible score) for each of the domains were: Symptoms: 18 (6–35), Fatigue: 33 (11–55), Emotional: 12 (3–15), Social: 32 (8–50) and Cognitive: 18 (6–30). Analyses were not adjusted for baseline characteristics. Statistical comparisons between pruritus severity categories were made where statistical power was ≥ 80% for a two‐sided significance of 0.05. PBC, primary biliary cholangitis.

Participants with any pruritus had significantly higher (worse) scores across all non‐itch PBC‐40 domains compared with those with no pruritus (Symptoms [19.04 vs. 14.05], Fatigue [37.55 vs. 26.82], Emotional [10.18 vs. 6.95], Social [32.34 vs. 20.77], Cognitive [18.03 vs. 14.14]; Figure 2). Participants with clinically significant pruritus had significantly higher (worse) scores across all non‐itch PBC‐40 domains compared with participants without clinically significant pruritus (Symptoms [20.76 vs. 16.66], Fatigue [41.7 vs. 31.8], Emotional [11.12 vs. 8.87], Social [36.4 vs. 26.68], Cognitive [20.11 vs. 15.15]) and greater odds of having clinically significant PBC‐40 domain scores versus those with no clinically significant pruritus (*p* < 0.0001; odds ratio [95% CI]: Symptoms 7.08 [3.89, 12.90]; Fatigue 9.51 [4.71, 19.22]; Emotional 2.67 [1.50, 4.76]; Social 6.14 [3.47, 10.88]; Cognitive 4.03 [2.33, 6.96]; Figures 2 and 3).

In the exploratory analysis, non‐itch PBC‐40 domain scores were significantly worse (all *p* < 0.0001) in participants reporting that itch sometimes, most of the time, or always disturbed their sleep versus those reporting itch never or rarely disturbs sleep (Table 3).

**TABLE 3:** Mean primary biliary cholangitis‐40 domain scores by frequency of pruritus‐related sleep disturbance.

PBC‐40 domain^a^	Itch never/rarely disturbs sleep (*n* = 86^b^)	Itch sometimes/most of the time/always disturbs sleep (*n* = 122^c^)	*p*
Mean ± SD			
Symptoms	16.91 ± 5.58	20.56 ± 4.95	*p* < 0.0001
Fatigue	32.29 ± 10.64	41.24 ± 7.96	*p* < 0.0001
Emotional	9.13 ± 3.28	10.97 ± 2.75	*p* < 0.0001
Social	27.56 ± 9.82	35.83 ± 8.36	*p* < 0.0001
Cognitive	15.55 ± 5.56	19.92 ± 5.58	*p* < 0.0001

*Note:* Higher PBC‐40 domain scores indicate worse outcomes.

Abbreviation: PBC, primary biliary cholangitis.

^a^ Thresholds for clinical significance (and the minimum and maximum possible score) for each of the domains were as follows: Fatigue: 33 (11–55), Cognitive: 18 (6–30), Symptoms: 18 (6–35), Social: 32 (8–50) and Emotional: 12 (3–15).

^b^ Emotional and Social, *n* = 85.

^c^ Emotional and Social, *n* = 121. Statistical comparisons between pruritus severity categories were made where statistical power was ≥ 80% for a two‐sided significance of 0.05. Analyses were not adjusted for baseline characteristics.

## 4 Discussion

These data utilizing the PicnicHealth PBC registry demonstrated that a significantly greater proportion of participants with high ALP levels had clinically significant pruritus than those with lower ALP levels. However, clinically significant pruritus was present across all ALP levels, and nearly a quarter of participants with normal ALP levels had clinically significant pruritus, indicating that patients with PBC can still suffer from debilitating pruritus despite PBC biochemical control. Additionally, a sensitivity analysis demonstrated that the association between ALP and itch severity was not significant. These findings underscore the importance of assessing all patients for itch regardless of other measures indicating disease control.

Participants with clinically significant pruritus, and those with more frequent pruritus‐related sleep disturbance, reported worse HRQoL across all non‐itch PBC‐40 domains (Symptoms, Fatigue, Emotional, Social and Cognitive) than those with non‐clinically significant pruritus and less frequent pruritus‐related sleep disturbance, respectively. Pruritus has previously been shown to be associated with sleep disturbance [2] and to substantially impact HRQoL [8, 9, 16, 17, 18]. Poor HRQoL can subsequently impact patients' social and career abilities, with patients with PBC and moderate/severe pruritus demonstrating worse social and cognitive PBC‐40 scores and worse Work Productivity and Activity Impairment scores than those with no or mild pruritus, as well as significantly worse scores for emotional reactions than healthy controls [6, 7]. Healthcare professionals should therefore focus on effective itch management in order to improve HRQoL, with the expectation that pruritus‐related sleep disturbance should then reduce. However, while reducing pruritus‐related sleep disturbance may be an important driver of improving HRQoL, the impact that PBC‐associated pruritus has on QoL is complex and multifactorial and may require a more holistic management approach.

This study utilized a real‐world population, reflecting the experiences and spectrum of patients with physician‐confirmed PBC outside of a controlled setting. The use of patient‐reported outcomes linked with medical records enables a deeper understanding of the interplay between clinical characteristics and PBC outcomes and the real‐life experiences of those living with PBC and PBC pruritus. The decentralized enrollment model, using different approaches for participant recruitment (i.e., PBC patient advocacy groups, affiliate partners and digital marketing campaigns), also maximized the opportunity for enrollment of patients with PBC representative of those seen in routine clinical practice. Pruritus is often under‐reported in commonly used observational data sources [3, 19] because there was no specific ICD‐10 code for cholestatic pruritus until 2024, meaning that pruritus experience can only be accurately captured through the use of patient‐reported outcomes. Hence, the PicnicHealth PBC registry dataset, which captures both patient‐reported outcome and medical record data, was appropriate for use in these analyses.

The use of the PicnicHealth PBC registry did however mean that the study population was limited; the majority of patients were White and neither Hispanic nor Latino which may not be representative of the entire US population. In addition, individuals with PBC who chose to enrol into the PicnicHealth PBC registry may reflect those who would benefit by gaining access to all their digitized medical records in one place, such as those with more severe disease or those with complex health needs and multiple records from many physicians. Finally, individuals enrolled may be those who are more motivated to participate in research, have experienced more symptoms than non‐participants, or been engaged with social media and patient advocacy groups; these factors may have resulted in selection bias. Furthermore, although other validated patient‐reported outcome measures are available (such as the worst itch numerical rating scale and 5‐D itch scale), the PicnicHealth PBC registry solely utilizes the PBC‐40 itch domain to categorize pruritus severity; it was selected as a disease‐specific QoL measure to capture multiple domains relevant to PBC while minimizing the burden of survey participation for respondents [20]. This study did not include total serum bile acid (TSBA) levels as an additional biochemical measure for pruritus since these were not consistently available in the medical records. One potential reason for this is that the measurement of serum TSBA levels is not part of current standard of care management of PBC [10, 21]. Furthermore, it is worth noting that serum TSBA levels do not always correlate well with pruritus severity [10, 22].

At the time of this study, some treatments were not yet labelled for use in PBC and therefore were not included within the analysis (e.g., peroxisome proliferator‐activated receptor [PPAR] agonists). Additionally, while some formal statistical comparisons were conducted as part of these analyses, others were necessarily descriptive due to a lack of power resulting from a small sample size. However, the trends observed in this rich dataset highlight the importance of holistic patient care and are worthy of further study. Finally, research with patient‐reported outcomes has the potential for recall bias or for poor recall; however, the PBC‐40 is a robust, validated tool for HRQoL measurement in PBC [5], helping to mitigate the issue.

In conclusion, while a greater proportion of participants with higher ALP levels had clinically significant pruritus compared with those with lower ALP levels, approximately one quarter of participants still experienced clinically significant pruritus despite normal ALP levels, highlighting that even patients with good PBC disease control may still require treatment for pruritus. Clinically significant pruritus is common in patients with PBC and significantly impacts their HRQoL, with pruritus‐related sleep disturbance also contributing to worsening HRQoL. Healthcare providers should assess every patient with PBC for pruritus and its impact on HRQoL, regardless of biochemical disease control and put in place a process of shared decision‐making with the individual patient for more holistic disease management, including treatment needs specifically for pruritus.

## Author Contributions

The authors meet criteria for authorship as recommended by the International Committee of Medical Journal Editors, take responsibility for the integrity of the work as a whole, contributed to the writing and reviewing of the manuscript and have given final approval for the version to be published. C.L., A.H., D.C.G., A.C., K.H. and K.B. contributed to the study concept/design. D.C.G., S.A. and H.S.F. contributed to data acquisition. D.C.G., D.P., H.V. and V.E.‐V. contributed data analysis. C.L., A.H., D.C.G., V.E.‐V., A.C., K.H. and K.B. contributed to data interpretation. All authors reviewed and approved the final manuscript.

## Funding

This study was funded by GSK (studies 218573 and 222959).

## Ethics Statement

This study was approved by the WCG Institutional Review Board (IRB Protocol ID: C‐PIC‐001; IRB Protocol #20214018). Participants gave informed consent to de‐identified research participation in the study, under the PicnicHealth IRB‐approved protocol and informed consent form, and for their medical records to be used in PicnicHealth's PBC registry. Therefore, further IRB approval, ethics committee review and informed consent were not required for this study. This study was conducted in accordance with the Declarations of Helsinki and Istanbul and complied with all applicable laws regarding participant privacy.

## Conflicts of Interest

C.L. has received research grants from Calliditas, Cymabay, Genfit, Gilead, GSK, Intercept, Ipsen, Kowa, Mirum, TARGET RWE and Zydus. A.C., D.C.G., D.P., K.H. and V.E.‐V. are employed by and hold financial equities in GSK. A.H. was employed by GSK at the time of the study, is currently employed by Novartis and holds financial equities in Novartis and GSK. H.V. is an employee of and holds financial equities in GSK. K.B. was an employee of GSK at the time of the study, employed through the Center of Health Outcomes, Policy and Economics at Rutgers University and is currently employed by Teva pharmaceuticals. S.A. is an employee of and holds financial equities in PicnicHealth. H.S.F. was employed by PicnicHealth at the time of the study, is currently employed by Sanofi and holds financial equities in PicnicHealth.

## Data Availability

Anonymized individual participant data and study documents can be requested for further research from www.clinicalstudydatarequest.com.
